# Orbital Plasmacytoma as Initial Presentation of Multiple Myeloma

**DOI:** 10.7759/cureus.108991

**Published:** 2026-05-16

**Authors:** Karla N Rodríguez Garcés, Martin F Picón Barrón, Leilani Gámez, Ulises Gomez-Alvarez, Claudia America Licea Moreno

**Affiliations:** 1 Internal Medicine, Hospital General Instituto de Seguridad y Servicios Sociales de los Trabajadores del Estado (ISSSTE) Querétaro, Querétaro, MEX; 2 Internal Medicine, Hospital General Instituto de Seguridad y Servicios Sociales de los Trabajadores del Estado (ISSSTE) Querétaro, Queretaro, MEX; 3 Hematology, Hospital General Instituto de Seguridad y Servicios Sociales de los Trabajadores del Estado (ISSSTE) Querétaro, Querétaro, MEX

**Keywords:** extramedullary multiple myeloma, immunohistochemistry, multiple myeloma, orbital plasmacytoma, plasmacytoma

## Abstract

Multiple myeloma (MM) is a hematologic malignancy characterized by clonal proliferation of plasma cells in the bone marrow. It represents approximately 10% of hematologic malignancies and is the most common primary bone malignancy. This disease usually occurs between the ages of 40 and 70, although it is more frequently seen in patients over 65 years of age. On the other hand, extramedullary plasmacytoma-related diseases have a global annual incidence of three per 100,000 people. Orbital involvement in MM is considered rare. Orbital plasmacytomas represent less than 1% of all orbital lesions, and their incidence in relation to MM is approximately 3%. Only 35% of cases present the initial manifestations of MM.

In the following case we present a 63-year-old male patient who presented with left ocular proptosis of one month’s duration, along with intermittent thoracic and lumbar back pain as well as weight loss of 12 kg in five months. A non-contrast cranial CT scan revealed a 31 x 31 x 36 mm extraconal orbital mass in the left orbit, with invasion of the paranasal sinuses and intracraneal extension, in addition to multiple lytic skull lesions. Laboratory results included severe normocytic normochromic anemia, mild thrombocytopenia, chronic kidney disease stage G3b, hypocalcemia, hyperphosphatemia, and elevated alkaline phosphatase. Immunohistochemistry demonstrated infiltration by clonal plasma cells (CD138 + and kappa +), findings consistent with MM.

## Introduction

Multiple myeloma (MM) is a malignant plasma cell neoplasm characterized by clonal proliferation of plasma cells, usually within the bone marrow. It is the second most common hematologic malignancy and typically affects older adults. Clinically, MM is commonly associated with anemia, renal impairment, calcium disturbances, and osteolytic bone disease, although its presentation may be heterogeneous and occasionally atypical. Early recognition is important because timely diagnosis and treatment may improve clinical outcomes and reduce end-organ damage [1-3].

Plasma cell neoplasms comprise a spectrum of disorders that includes MM, solitary bone plasmacytoma, and extramedullary plasmacytoma. In MM, plasma cell proliferation is usually systemic and marrow-based, whereas solitary plasmacytomas represent localized tumors without evidence of diffuse systemic disease at the time of diagnosis. Extramedullary plasmacytomas arise in soft tissues, most commonly in the upper aerodigestive tract. Distinguishing these entities is clinically relevant because they differ in diagnostic workup, treatment strategies, and prognosis [4-6].

Orbital involvement by plasma cell neoplasms is rare. It may occur either as an isolated extramedullary plasmacytoma or as a manifestation of systemic MM. Reported ophthalmologic manifestations include proptosis, diplopia, ptosis, ocular pain, visual impairment, and other signs related to orbital mass effect. Because these findings are nonspecific and may mimic other orbital tumors or inflammatory processes, diagnosis may be delayed, particularly in patients without a previous hematologic diagnosis [7-12].

Although uncommon, orbital involvement may represent the first clinical manifestation of an underlying plasma cell dyscrasia. For this reason, it should be considered in the differential diagnosis of atypical orbital masses, especially when accompanied by systemic findings suggestive of hematologic disease. Herein, we report a rare case of orbital involvement as the initial manifestation of previously undiagnosed MM, highlighting the importance of maintaining a high index of suspicion and performing prompt systemic evaluation in such patients [8,10-14].

## Case presentation

A 63-year-old male patient with no known previous comorbidities presented with left ocular proptosis of less than one month’s duration. He also reported chronic lumbar pain and unintentional weight loss of approximately 12 kilograms over five months.

On initial ophthalmologic evaluation, left ocular proptosis was confirmed. Best-corrected visual acuity was 20/20 in the right eye and 20/200 in the left eye. Extraocular motility was restricted, with associated diplopia. Pupillary light reflex was preserved. Intraocular pressure was 18 mmHg in both eyes, and funduscopic examination showed no evident abnormalities.

**Figure 1:**
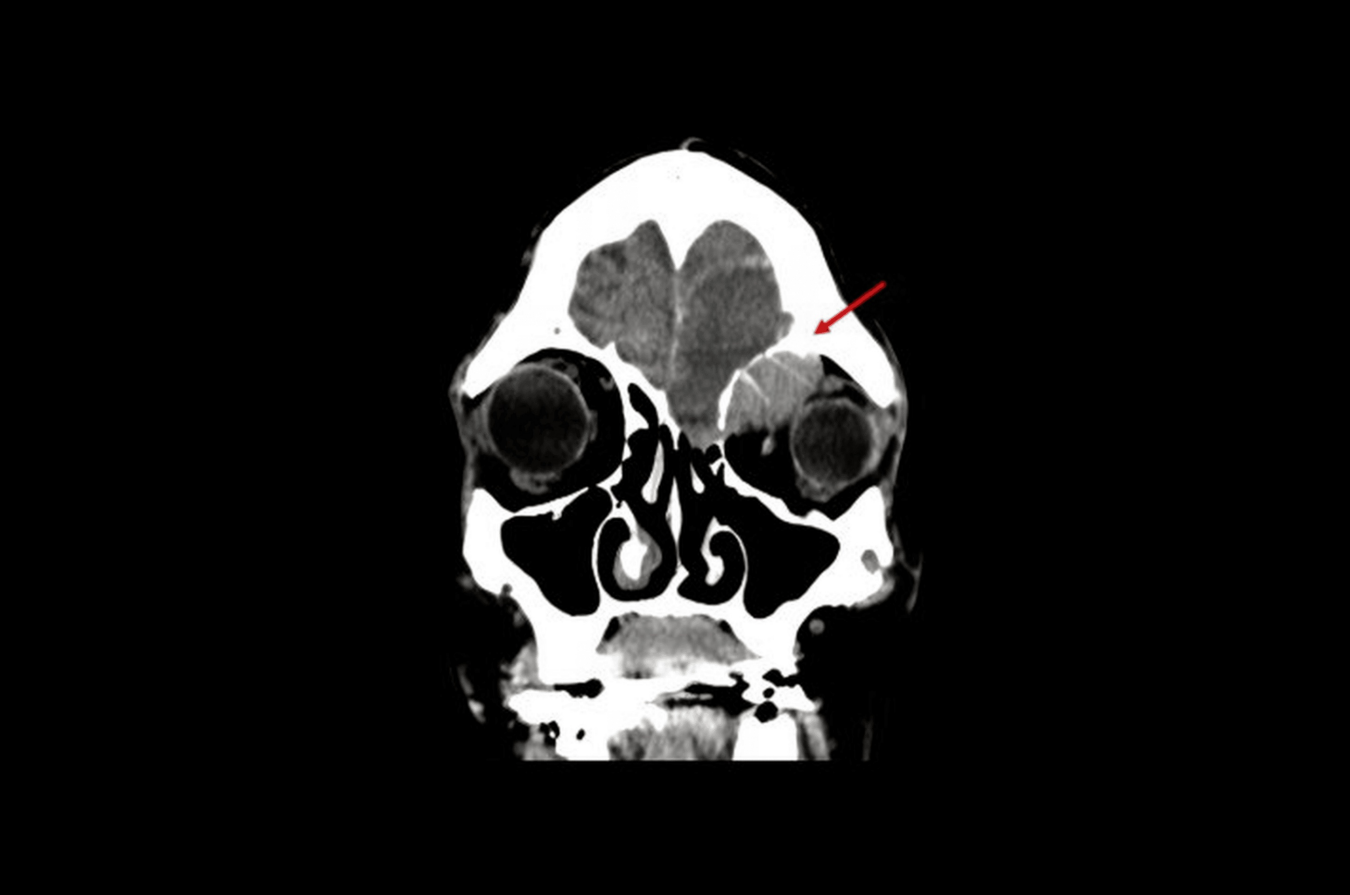
Cranial and paranasal sinuses CT scan, coronal section Coronal CT scan showing a heterogeneous extraconal soft tissue lesion in the left orbital cavity (31 × 31 × 36 mm), located in the superolateral quadrant, with poorly defined margins and significant inferomedial displacement of the ipsilateral globe, consistent with proptosis.

A non-contrast cranial CT scan was performed as the initial imaging study and revealed a 31 × 31 × 36 mm extraconal mass in the left orbital cavity, displacing the ipsilateral globe, with loss of architecture of the left frontal and ethmoidal sinuses and extension into the cranial cavity (Figure 1). Multiple lytic lesions were also identified in the skull. Given these findings, further evaluation was undertaken to investigate the extent of disease.

**Figure 2:**
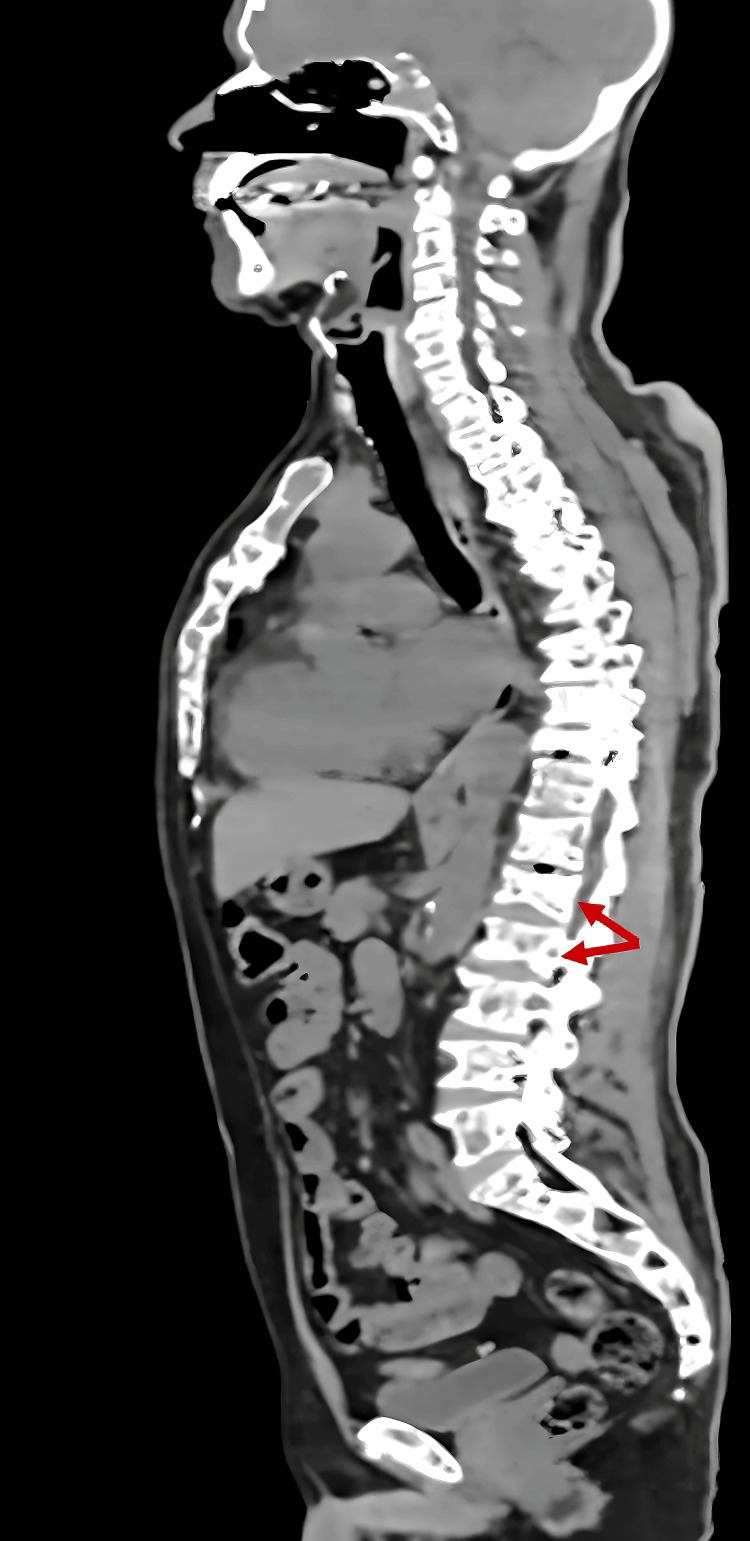
Thoracoabdominal CT scan, sagittal plane, bone window Sagittal CT scan (bone window) of the thoracoabdominal spine demonstrating multiple osteolytic lesions throughout the axial skeleton, with loss of vertebral body height at several levels, most notably at the thoracolumbar junction (arrowhead), suggestive of pathological compression fractures.

Subsequent non-contrast thoracic and abdominal CT scans revealed compression fractures of the T6 and T7 vertebral bodies, multiple lytic lesions in the axial and appendicular skeleton, and multilevel discopathy (Figure 2). These imaging findings, together with the patient’s constitutional symptoms and bone pain, raised suspicion of an underlying plasma cell neoplasm.

Biochemical laboratory tests, obtained sequentially during the diagnostic workup, showed severe normocytic normochromic anemia, with hemoglobin of 5.4 g/dL and hematocrit of 18.4%; thrombocytopenia, with a platelet count of 83,000/mm³; renal dysfunction, with creatinine of 3.4 mg/dL; calcium alteration, with calcium of 7.8 mg/dL; and phosphorus alteration, with phosphorus of 2 mg/dL.

**Figure 3:**
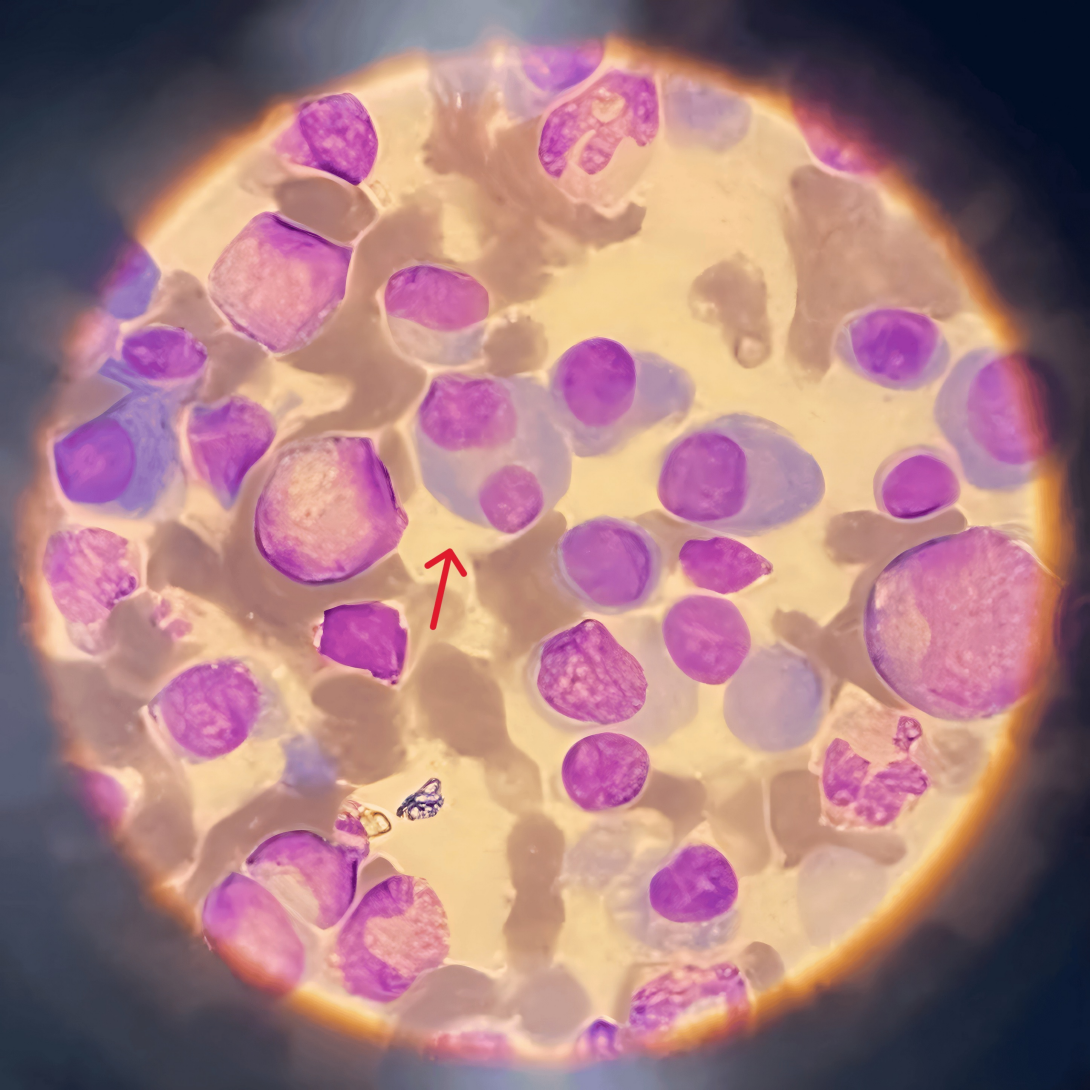
Wright-Giemsa-Stained Bone Marrow Aspirate Bone marrow aspirate (Wright-Giemsa stain) demonstrating a marked predominance of neoplastic plasma cells of variable size, with abundant pale cytoplasm and eccentric nuclei. A red arrow highlights a binucleated plasmacyte showing two distinct nuclear profiles, a hallmark of malignant plasmacytic proliferation consistent with multiple myeloma.

In view of these systemic findings, a bone marrow aspirate was performed and revealed a predominance of plasma cells with binucleated and multinucleated forms, consistent with neoplastic plasma cells. Immunohistochemical analysis of the bone marrow specimen demonstrated a clonal plasma cell population positive for CD138 and kappa light chains, supporting the diagnosis of a plasma cell neoplasm in the context of MM (Figure 3). No biopsy of the orbital mass was performed. Therefore, the diagnosis was established on the basis of imaging findings, systemic clinical and laboratory abnormalities, bone marrow aspirate morphology, and immunohistochemical demonstration of clonal plasma cells in the bone marrow.

The patient was subsequently referred to the Hematology Department, where treatment with VRd therapy (bortezomib, lenalidomide, and dexamethasone) was initiated. He showed good initial tolerance and progressive clinical improvement, including reduction of proptosis. Throughout the induction cycles, no peripheral neuropathy, gastrointestinal toxicity, thromboembolic events, or infectious complications were observed. The patient completed the planned cycles without dose reductions or treatment interruptions, reflecting a favorable tolerability profile. Autologous hematopoietic stem cell transplantation was planned as consolidative therapy.

## Discussion

MM accounts for approximately 10% of hematologic malignancies and is one of the most common plasma cell neoplasms in older adults. Although it usually presents with systemic manifestations, extramedullary disease may occur, and orbital involvement is considered uncommon [1,7-9]. Reported orbital manifestations include proptosis, decreased visual acuity, diplopia, ptosis, ocular pain, and limitation of extraocular movements. In some patients, these findings may represent the first manifestation of otherwise unrecognized systemic disease [8,13-15].

The present case is clinically relevant because the patient initially presented with unilateral proptosis and visual symptoms, while subsequent evaluation revealed multiple osteolytic lesions, severe anemia, renal dysfunction, and bone marrow infiltration by clonal plasma cells. This combination of orbital and systemic findings was essential in raising suspicion of MM as the underlying diagnosis. Similar presentations have been described in previously reported cases of orbital involvement associated with MM [3-5,13-15].

The differential diagnosis of an orbital mass with unilateral proptosis includes inflammatory, lymphoproliferative, and other neoplastic conditions, such as orbital lymphoma, inflammatory pseudotumor, metastatic disease, meningioma, lacrimal gland tumors, and other primary orbital masses. In the present case, however, the coexistence of a destructive orbital lesion with skull involvement, multiple osteolytic lesions, constitutional symptoms, anemia, and renal dysfunction made a plasma cell neoplasm a major diagnostic consideration [7,13-15].

Histopathologic confirmation of extramedullary plasmacytoma is ideally obtained from the involved soft tissue [16]. In the present case, no orbital biopsy was performed. Instead, the diagnosis was supported by the clinicoradiologic findings together with bone marrow aspirate morphology showing neoplastic plasma cells and immunohistochemical analysis of the bone marrow specimen demonstrating CD138-positive, kappa-restricted clonal plasma cells, consistent with MM [17,18].

Because this patient had evidence of systemic disease rather than an isolated extramedullary lesion, treatment was directed at MM. Induction therapy with VRd was initiated, as this regimen is commonly used in newly diagnosed MM and allows disease control before consolidation. In view of the patient’s overall clinical status and planned transplant-oriented management, autologous hematopoietic stem cell transplantation was considered as consolidative therapy [19,20].

## Conclusions

Although MM usually presents with systemic manifestations, orbital involvement as the initial presentation is rare and clinically significant. This case highlights the importance of considering MM in patients with atypical orbital findings, particularly proptosis, when accompanied by systemic warning signs such as weight loss, bone pain, anemia, renal dysfunction, or osteolytic lesions. It also underscores the need to integrate ophthalmologic examination with imaging, laboratory, and bone marrow findings in order to establish the diagnosis in unusual presentations. In this patient, recognition of the underlying disease allowed for initiation of VRd induction therapy, with favorable initial clinical response. A multidisciplinary approach remains essential in such cases to ensure appropriate diagnostic workup and timely treatment of what may otherwise be a diagnostically elusive condition. 
